# Reflex Irritations Throughout the Genito-Urinary Tract

**Published:** 1875-05

**Authors:** 


*Reflex Irritations Throughout the Genito-Urinary Tract*, resulting from contraction of the Urethra at or near the Meatus Urinarius, Congenital or Acquired. By Fessender N. Otis, M. D. Clinical Prof. of Genito Urinary Diseases in the College of Physicians and Surgeons, New York. Published by McDevitt, Campbell & Co., 111 Nassau St., N. Y., 1875.

